# How adolescent motherhood is perceived and influenced by sociocultural factors: A sociological qualitative study of Sindh province, Pakistan

**DOI:** 10.1371/journal.pone.0319064

**Published:** 2025-03-31

**Authors:** Nadia Agha, Rahim Dad Rind

**Affiliations:** 1 Department of Sociology & Gender Studies, Shah Abdul Latif University, Khairpur, Pakistan; 2 Department of Sociology, University of Sindh, Jamshoro, Pakistan; Torrens University Australia, NEPAL

## Abstract

**Background:**

In Low and Middle-Income Countries (LMICs), 21 million adolescent girls aged between 15 and 19 years become pregnant each year. Adolescent girls in South Asia have the shortest interval to begin maternity, i.e., within 1–2 years of marriage. There is limited evidence on the associated factors of adolescent pregnancy and motherhood in rural areas where socioeconomic indicators about women are weak. This study examined the sociocultural factors intertwined together and gave rise to adolescent motherhood.

**Methods:**

This exploratory qualitative study was conducted in rural and less privileged areas in Pakistan’s Sindh province. Data was collected from August 05 to September 17, 2023, and 37 young women, who experienced early marriage, adolescent pregnancy and motherhood, were interviewed. The data was translated and transcribed verbatim. Braun & Clark’s six-step data analysis Model was used to create codes, develop themes and generate a report.

**Results:**

The findings of the study show that child and close kin group marriage, poor socioeconomic conditions and social norms encouraged adolescent motherhood among young women. One’s association with the extended family also increased the likelihood of early maternity because different family members exerted social pressure to become a mother. This made the girls anxious to begin motherhood soon after marriage. However, these young mothers were unaware of adolescent’s motherhood’s social, psychological and physical consequences. They embraced it happily because it was considered a way of strengthening their position in the family.

**Conclusion:**

This study confirms the negative consequences of adolescent motherhood in terms of school dropout and poor health outcomes. Based on these findings, we suggest addressing poverty and female dropout rates to prevent child marriage and adolescent pregnancy. Health providers must be trained to provide awareness and psychological support to girls experiencing adolescent motherhood. The government must initiate sexual and reproductive health education and engage community heads and religious leaders to educate communities about the social and health consequences of child marriage, adolescent pregnancy and motherhood.

## 1. Introduction

Adolescent motherhood is a global public health concern prevailing in many countries worldwide [1]. Around 13 to 17 million women under the age of 20 gave birth to their first baby in 2020, 11% of total births globally [2]. This causes an increased risk of adverse obstetric and health consequences for women and their children such as puerperal endometriosis, eclampsia, preterm birth, low birth weight and other complications [3,4]. Nutritional anaemia is one of the major health problems during pregnancy [5]. Girls with adolescent pregnancy have limited access to educational attainment, which ultimately deprives them of economic opportunities [6–9]. Therefore, the prevention of adolescent pregnancy and child marriage – the root cause of adolescent motherhood – has gained more attention in the world. This issue has also been embedded in the United Nation’s Sustainable Development Goals agenda [4].

### 1.1. Adolescent motherhood in Low and Middle-Income Countries

In Low and Middle-Income Countries (LMICs), early marriage and adolescent motherhood is one of the significant health challenges and has a greater impact on mothers, children, families and communities. In LMICs, 21 million adolescent girls aged between 15–19 years become pregnant each year, around 50% of which were unintended pregnancies [10]. Several intertwined factors lead to adolescent motherhood, e.g., child marriage, lack of sex education, peer pressure, family experience of adolescent birth, healthcare services and so on [11].

South Asia continues to have one of the highest rates of adolescent pregnancy, with a 35% prevalence rate in Bangladesh, 21% in Nepal and 21% in India [12]. Socio-demographic and cultural factors like limited female literacy, lower socio-economic status and being an ethnic or religious minority give rise to the prevalence of early marriage and adolescent pregnancy [13]. For example, women belonging to scheduled caste in India have higher odds of early maternity than their general caste counterparts [14]. The economic status of women and adolescent pregnancy are significantly associated with each other. Girls with low economic status and income are more prone to be pregnant at an early stage of their lives than those with higher economic backgrounds [15]. Lack of economic opportunities, limited access to quality education, and lack of information about sexual and reproductive health and rights further exacerbate the situation for young women living in South Asian countries. There is limited evidence on the associated factors of adolescent pregnancy and motherhood in rural areas where women are in a disadvantaged position. Despite the multiple measures and interventions of different stakeholders and governments, adolescent motherhood continues to be prevalent in LMICs, including Pakistan, where child marriage is mainly responsible for adolescent pregnancy and motherhood [16–18].

### 1.2. Child marriage and adolescent motherhood

Child marriage, especially in LMICs, is one of the most pressing human rights issues today. The practice of child marriage has a significant impact on girls’ and women’s later life trajectories [19]. Therefore, child marriage is now an integral part of the global development agenda due to its inclusion in Sustainable Development Goal (SDG) 5.3, which seeks to eliminate child marriage by 2030.

South Asia has one of the highest incidences of girl-child marriage (44%), and India has one-third of the global total of these marriages [20]. It is estimated that South Asia contains about 285 million women marrying before age 18; about one in four young women in South Asia was married or entered into a partnership before turning eighteen. The percentage of child brides is highest in Bangladesh (59%), followed by Nepal (40%) and Afghanistan (35%). Pakistan ranks sixth globally, where 21% girls are married before 18 years [18]. This practice has a devastating impact on girls’ education, as a vast majority of child brides drop out of school [21].

There are various factors influencing child marriage: low literacy among girls and parents, economic issues, gender norms and gender-based violence [22]. Social norms limiting women’s autonomy and rights, religious beliefs and the preservation of the family’s honour also influence child marriage [23]. Some communities in Pakistan use marriage as a safeguard against pre-marital relationships and sexual assault, as well as a means of upholding the family’s honour. Child marriage is also viewed as a way to lessen the family’s financial burden [24]. The availability of prospective grooms who belong to a specific caste and religion, the economic wealth of the groom’s family, sound educational credentials, and the provision of finances for dowry are contributing factors to child marriage [25]. More specifically, the prevalence of cultural and traditional practices, poverty, lack of awareness, limited literacy and social insecurity encourage child marriage in Pakistan [26].

The northern region of the Sindh province is often highlighted with the number of child marriage cases. Districts Jacobabad and Kashmore in northern Sindh are considered hotspots with the highest incidence of child marriage cases, with the district of Jacobabad topping the list in child marriage under 15 years. It is estimated that 14 out of 29 districts in Sindh have witnessed a rise in the incidence of child marriage [17]. Child marriage, typically arranged by parents, is more common among girls from low-income and low-education households [27]. The Decision-making mandate concerning children’s marriage in Sindh rests with parents [28]. Young adolescents, more particularly women, are unable to make any decisions about their lives because of the patriarchal setup where fathers’ and older men’s decisions prevail. Kakal et al.’s [28] study further reveals that the educational status of both children and parents has a greater influence on the likelihood of child marriage in the family. Gender discrimination, lack of quality educational and economic opportunities are contributing factors to child marriage in Sindh. Rural communities in Sindh are often aware of the adverse impact of child marriage on women’s health. However, women are still encouraged to engage in childbearing [16]. This eventually leads to adolescent pregnancy and early childbearing.

Child marriage, teenage pregnancy and adolescent motherhood carry serious health, social and economic challenges for young women [29]. The high number of adolescent mothers in South Asia is the direct consequence of child marriage [30]. Although child marriage has witnessed a decline over time, it has not been eliminated and is common in many parts of the world. It is estimated that around 650 million women worldwide were married in adolescence, many of these women (around 60 million) live in West and Central Africa [31]. Child marriage increases women’s vulnerability to resist violence and harmful practices. It is also a barrier to education, which is linked to delaying child marriage [32]. Practices like child marriage, embedded in local culture, expose young girls to early maternity and further restrict the flow of educational, employment and health opportunities to them [33].

Prevention of pregnancy and mortality and morbidity among adolescent girls during pregnancy is fundamental to achieving positive health outcomes. It is imperative to achieve the Sustainable Development Goals (SDGs) associated with maternal and newborn health [29]. The WHO declared some indicators to improve maternal health, which aim to remove all harmful practices including child marriage, around the world by 2030 [34]. Despite all of this, adolescent motherhood is still highly prevalent globally.

In Pakistan, growing attention has been paid to improving access to quality maternal healthcare for pregnant and parenting adolescents. Pakistan has also pledged on different forums to eliminate child marriage. For instance, the country affirmed ending child marriage on the South Asian Association for Regional Cooperation (SAARC) platform “Kathmandu Call to Action to End Child Marriage”. Pakistan also agreed to accept Beijing’s Declaration and Platform for Action. Despite all these commitments, the pervasive occurrence of child marriage remains one of the grave concerns for the country [35]. This research examined the sociocultural factors intertwined together and gave rise to adolescent motherhood. This study aims to explore how sociocultural factors induce adolescent motherhood and how cultural practices and extended family systems further influence young girls to conform to those practices. Particularly in the context of rural Sindh, where the rigid form of patriarchy and cultural practices prevail, there is limited evidence on child marriage and adolescent motherhood based on the first-hand experiences of the women affected by such practices.

## 2. Materials and methods

### 2.1. Study design, procedure and participants

This is an exploratory qualitative study. It was essential to follow a qualitative research design to answer the research question, i.e., how sociocultural factors influence adolescent pregnancy and motherhood in a rural setting. The fieldwork for this study was conducted in six villages of northern Sindh in southern Pakistan by a disciplinary team under the supervision of the first and second author. Specific needs, proximity, budget and time frame guided the decision to select the villages. A vast majority of people in these villages were engaged in agricultural activities, providing them a source of livelihood. The villages had inadequate healthcare facilities and villagers would visit the district headquarters to access secondary and tertiary care. However, there was a Rural Health Centre by the Government of Sindh providing maternal and child health facilities close to each village. These healthcare centers were situated 2-3 kilometers from each village and lacked quality services.

The participants’ recruitment process and data collection started from August 05 to September 17, 2023. A total of 37 women aged between 15 to 20 years were purposively recruited who met the inclusion criteria, i.e., those who belonged to rural and less privileged areas, had child marriage and experienced adolescent pregnancy and childbearing within two years of postpartum. Documenting the experiences of pregnancy and childbirth within two years of postpartum enabled us to analyze a recent picture of ongoing local practices and their implications for young girls. Therefore, the women who were married after 18 years, did not experience adolescent pregnancy or did not deliver a baby within two years of data collection were excluded from the sample. A woman who had given stillbirth within two years was also included in the sample because of her pregnancy and childbirth experience. We understand that including men as study participants would have provided enriching data and insight into their role in encouraging early motherhood. However, this study focused on young women and older women’s influence on them. In the cultural context under study, young women are reluctant to discuss reproductive and sexual health matters with men, women-to-women conversations on such matters are more frequent. Thus, young women are more influenced by older women than men. Secondly, this study covered several areas, including social and cultural practices and women’s life challenges. Gaining rich data on these issues was challenging and time-consuming because some participants were shy and less responsive. Therefore, we studied a comparatively larger sample size and continued to look for new information until we reached data saturation. For these reasons, we restricted data collection to young women.

### 2.2. Ethics

This study was approved by the Institutional Ethical Review Board (IERB) at Shah Abdul Latif University, Khairpur (Reference No.ORIC./SALU//KHP/301, dated July 31, 2023). All ethical procedures mentioned in the proposal were followed. It is worth mentioning here that the formal age of consent for females in Pakistan is 16 years. Sindh is the only province that has passed legislation to make the legal age of marriage 18 years for both males and females. Yet, many young girls in rural and remote areas are married before this legal age because their guardian/fathers are culturally authorized to make decisions on their behalf. This was also discussed with the IERB and it was resolved that guardians of the participants will be involved throughout the data collection process. Therefore, the authors first met the villagers, introduced themselves and the aims and objectives of the study, and sought formal verbal permission from the family heads of the participants to meet and interview the participants. These family heads were usually the participants’ fathers – in – law/guardians and husbands.

Later, the aims and objectives of the study were explained to all the participants and the verbal consent was recorded in the presence of the first author, who was also supervising the research team. Ethical concerns regarding confidentiality, anonymity and willingness of the participants to be interviewed were thoroughly discussed and approved by the IERB. Thus, all identifiable information has been anonymized, the participants’ identities have been kept confidential, and their anonymized names have been used throughout this paper.

Most participants lived in extended families where maintaining privacy during interviews was difficult. Therefore, we requested privacy from the guardians and family members to provide a comfortable environment for the participants. In most of the houses, the interviews were conducted in separate rooms so the participants could speak without any disturbance or fear. In the houses where no separate room was available, the interviews were conducted in the courtyard. The family members left the courtyard to provide privacy.

Accessing the research subjects in a rural context was challenging. The authors’ positionality was likely to influence every stage of the research; however, addressing this positionality allowed for the ethical conduct of this study. For instance, the authors’ education level, employment and status could create biases and power imbalance. Nevertheless, the researchers ensured that the participants were free to answer the questions and withdraw from the interview at any moment. Moreover, the authors’ ethnicity, proficiency in the local language, informal conversations with families and participants, use of local attire, residence in a nearby area, the first author’s gender and their positions in a government sector university enabled them to gain the trust of the family heads and maintain rapport with the participants. The villagers particularly paid respect to women and were hospitable to their guests.

### 2.3. Data collection

We conducted face-to-face in-depth interviews for this study to collect insightful data. The duration of each interview varied depending on how vocal a participant was. For instance, the participants who gave short answers took ten minutes for an interview whereas the maximum time spent was 20 minutes. All interviews were audio recorded and conducted at the participant’s house by the female interviewers in the disciplinary team. The interview guide was developed in Sindhi considering the questions and issues to be explored. A review of relevant studies helped to improve and refine it further. The interview guide was comprised two sections: the first section carried demographic characteristics, marriage arrangements and pregnancy-related information. The second section carried open-ended questions on marriage, pregnancy, and delivery. For example, questions included how the marriage was arranged, under what circumstances the marriage was contracted, what changes the participants experienced after the marriage at a young age, what forms of social pressures the girls faced after marriage, how they dealt with the social pressure for getting pregnant, what was the outcome of those pressures, how the participants felt upon getting pregnant, how they carried prenatal and antenatal care, what support the participants received from their family members etc.

We understand that social research establishes a seemingly balanced relationship between the researcher and the researched, but the situation is different in practice [36,37]. Initially, we assumed that dealing with young participants with limited or no literacy and restricted access to the social sphere would make it easier for us to extract information. We had also assumed that women would provide clear and detailed answers to the researchers who were natives. However, exploring the impact of the sociocultural practices that consciously or unconsciously exploit women’s well-being was challenging; for example, there were moments when the participants did not speak to the research team at length or tried to avoid giving detailed answers. We then had to think through the ways that could allow us a better understanding of the situation and how our potential power as researchers was challenging to us. We had to work harder to build rapport with the respondents’ families and familiarize with the participants to bring them to the point where they spoke to us at length. Secondly, it also took us longer to explore how women negotiated with patriarchy while they accepted those sociocultural practices and did what was expected of them. Deep inside them, they tolerated all this and embraced cultural practices in the hope of better days when they would have children to strengthen their position in the family.

### 2.4. Data analysis

We anonymized all identifiable information for data analysis, including the participants’ names. All data collection team members were natives and had command over both languages, i.e., Sindhi and English. Therefore, the data was transcribed and translated simultaneously from Sindhi to English. A six-step thematic analysis model suggested by Braun & Clarke was followed for the data analysis procedure [38]. The model helped to understand the data and develop the themes. For example, we first familiarized ourselves with the data, generated codes and collated the codes with supporting data, which led to generating themes, e.g., the first emerging theme was ‘Determinants of marriage’. We then reviewed and refined the themes and generated the report. This six-step process helped significantly to answer the research question.

## 3. Findings

The respondents in this study married young and started childbearing in adolescence (between 15 and 16 years). Many young women either had limited or no literacy and lacked basic information and awareness about reproductive health, antenatal check-ups, and contraceptive use. Socio-demographic details of the respondents are given in Table 1.

**Table 1:** Socio-demographic details of the participants (n = 37).

	Frequency	Percentage
**Age of the respondents**		
15–16	09	24%
17–18	08	22%
19–20	20	54%
**Age at marriage**		
13–14	07	19%
15–16	22	59%
17–18	08	22%
**Age at the first pregnancy**		
15–16	20	54%
17–18	17	46%
**No of children**		
None	01	3%
01	08	22%
02	22	59%
03	06	16%
**Respondents’ education level**		
None	16	43%
Primary	08	22%
Secondary	03	8%
Higher Secondary	02	5%
Intermediate	06	16%
Graduate	02	5%

The interviews with the participants and the Model used for data analysis revealed three main themes: (1) Determinants of marriage; (2) Social pressures and early motherhood; and (3) Impact of social pressure. These themes helped to answer the research question; for example, the first theme explains marriage arrangements in the villages and what encouraged parents to marry off their daughters at a young age. This led to building an argument about how poverty and limited access to economic opportunities accelerate the incidence of child marriage and adolescent motherhood.

### 3.1. Determinants of marriage

Most of the participants in this study married between 15 – 16 years. The main determinant of their marriage was the onset of puberty and the availability of a potential groom within the close kin group. Endogamous marriage was common in the villages and enabled parents to find a potential groom for their daughters. Adolescent girls had no say in marriage decision-making since parents and elders arranged marriages in the villages. Both the bride and the groom know from their childhood whom they will marry. Saima, married at the age of 17 to her cousin, said:

“*My parents stressed my marriage when I started menstruating. I am married to my cousin, he was earning so my parents immediately agreed. It is not a fairy tale but the same common story of every girl like me.”*

Kin group marriage also facilitates the patriarchal control of a girl’s life since her childhood. In-laws can direct the parents of a girls about their wishes. For example, Tahira, who was married with her first cousin at the age of 15, was forced to leave education because her fiancé did not consider it good for her. He convinced his parents to talk to Tahira’s parents to discontinue her education. Later, Tahira was forced to quit her primary schooling.

Unfortunately, such acts are not considered unusual or a violation of rights either by girls or their families. The prevalent cultural practices and norms made the young women internalize the system of early marriage and consider this a usual practice followed by all girls. Every rule or desire of the elders that came along with marriage was accepted without resistance. Rabia, who had a cousin marriage at the age of 15, shared:

*“There is no pressure on girls for marriage because early marriage is common here. My parents selected my husband and I agreed.”*

Poor socioeconomic conditions of a family, such as restricted access to financial, social, educational and health resources, made education unaffordable and irrelevant to marriage for girls. Marriage was essential, and parents were urged to contractually organize their daughter’s marriage as soon as they found a suitable match. It was a prevalent belief that daughters have their permanent residence at their husband’s houses, where they could afford their financial needs and health expenses, so they should leave for their permanent abodes as soon as possible. Nisa, who belonged to a poor family and was married at the age of 16, said:

*“We are four sisters and one brother. My father had a humble job and had a hard time raising us. Education was unaffordable, so we discontinued it. He then started looking for potential grooms for his daughters. Someone told him about two brothers who were labourers and had a good reputation. My father agreed and married his two daughters at a young age.”*

Child marriage for dispute settlement was also used in the villages. Usually, a dispute can be settled after a penalty is paid by the person or his family who has committed a crime or an offence. However, when a family is poor, it cannot pay a fine for settling the dispute with another family. Thus, the exchange of young daughters in marriage is used to de-escalate the conflict. Seema had to be married at 13 when her married brother eloped with another girl. Seema was then married in exchange for a dispute settlement to teach her brother a lesson. In this way, her parents not only resolved the family issue but also fulfilled the responsibility of marrying the daughter.

Social pressures on parents to marry their daughters do not stand alone. Poverty, lack of social security, and support from close relatives often urge parents to hand over their daughters to the men who could protect them. Marriage is considered the most critical factor in strengthening a social network with the family of the groom. This support is significant in easing financial pressure on parents.

### 3.2. Social pressures and adolescent motherhood

The societal expectations placed on girls to marry do not cease after they wed but evolve into new forms. The child brides in this study began motherhood at an early age even before they knew about their own sexual and reproductive health. In this process, the extended family played a vital role in exercising pressure on the girls and conforming them to their reproductive role. Most of the participants lived in extended families with their father–in–law, mother–in–law, husband’s brothers, their wives and children. This enabled women’s regular close contact with each other. This also made it difficult to maintain privacy in their personal lives. Thus, other women in the family would repeatedly enquire about young married girls and whether they had any ‘good news’. The participants were compelled to get pregnant and strengthen their position in the family. Nazia, who was married at the age of 15 to her cousin, shared the common practice in her village:

*“I got married at 15. I did not know anything about being married or making a home. In my village, young married girls live under pressure from in-laws and other older women in the family. I was under pressure in the first two months of my marriage as I did not see any signs of getting pregnant, but thanks to God, I was relieved when I conceived after two months.”*

The social pressures that young girls encounter vary in nature. For example, a common perception in the villages is that motherhood strengthens a woman’s position in the husband’s family and ensures her permanent stay. The women who bear more sons, preferably through normal delivery, and do household work are considered ideal women and more satisfying to men. This reduces men’s chances of a second marriage. Therefore, the more satisfying a woman is, the lesser the chances of her husband indulging in polygamy. Saima, who was married at 17, said:

*“In our community, young girls get pregnant soon after marriage. If they do not get pregnant, their husband could be married to another girl to get children. We are easily misled because we fear our husband’s second marriage.”*

Another participant, 19–year–old Tahira, who was married at the age of 15, told how she experienced a challenging situation when women from her neighbourhood and kin group started enquiring about her pregnancy. She said:

*“It usually becomes a concern of everyone after 2-3 months of the marriage of a girl. Whenever any woman from my neighbourhood visited my home, she first inquired me about pregnancy. I have been told since the first day that homemaking is the sole purpose of a girl’s life. My mother told me that I would have a respectable position in my in-law’s house after childbearing. From both fronts, I was guided to get pregnant as soon as possible.”*

Young married girls in the villages faced different forms of social pressures to adjust to their in-laws and do as they pleased. The participants shared that the relatives and family members also expected a male child from them, along with other expectations. 20–year–old Nazia shared that she experienced severe stress because everybody in the house expected a baby boy. Some women in this study were second wives and were married solely for a male child. Since the kinship system in the villages was patrilineal and the descent was traced through the male line, it was essential for a man to have sons. Therefore, polygamy was acceptable in the villages if a man did not have a male child. Seema, who was the second wife of her husband and was married at the age of 13, said:

*“I was too young when I got married. In the second month, I got pregnant and I faced pressure to deliver a son because it was my husband’s second marriage. He did not have a male child from his first marriage. I was afraid that if I was unable to produce a son, he would beat me.”*

Another participant, Saima, married at 17 to her cousin, who was the only brother of six sisters. There was no man in the family other than her husband, which was why her mother and sisters–in–law would repeatedly tell her their wishes for a male child. Saima said:

*“Whoever I met after my marriage, I was asked why I was late in getting pregnant. I would often go into stress thinking almost every moment when would I get pregnant. When I would get spotting, I was scared, considering it periods. In my case, I was forced and conditioned in a way that my husband has six sisters. He is the sole brother to them. I was asked to bless them with a baby boy now.”*

### 3.3. Impact of social pressure

Social pressures exercised on young girls in the villages to become mothers were immense and had its repercussions. The participants shared that the questions inquiring about their pregnancy echoed in their minds for several days and caused distress. The criticism they faced kept them disturbed mentally till they conceived. Saima continued:

*“Young married girls in my village often go into depression, thinking almost every moment when would they get pregnant. I used to get down in despair and hopelessness: whenever I heard questions enquiring about pregnancy, I would leave eating and cry in some lone corners.”*

Another participant, 19–year–old Nisa, who was married at 16, said:

*“When I did not conceive in the initial months of my marriage, my insecurity and fears increased. Sometimes, I overheard people saying I would not be able to give birth to a child. I stopped attending social events to avoid people.”*

Sofia was just 16 at her marriage. She did not conceive in the initial few months of her marriage. Repeated inquiries about her pregnancy were embarrassing and had put her under pressure. She spent many sleepless nights crying as she was unable to meet the expectations of her community members, particularly her mother-in-law. She said:

*“Social pressure on me was extreme. I had to convince my husband as he was not ready for a child. I told him that I wanted to become a mother as I was under pressure from family and community, which I was unable to face. I got pregnant after three months of my marriage.”*

The young women lived in insecurity and doubt until they succeeded in conceiving and providing evidence of their fertility, capacity to reproduce and being able to provide an heir to the family. This single moment was enough to make the participants forget all the worries and tensions they faced before getting pregnant. Many women said that they felt safe and secure after getting pregnant. Seema, who went through many stressful months, shared with happiness:

*“I felt secure and proud to be pregnant. Although financial problems made me mentally disturbed, the gains women make through giving birth are larger than anything else. They secure their future and permanent stay at their husband’s house.”*

To reach this position, the girls had sacrificed unconsciously. For example, some of the girls had to leave schooling after getting pregnant, while others had to deal with deteriorating health conditions of pregnancy, which limited their access to schools. A few respondents had to remain at home to perform house chores and could not cope with the burden of household work, which did not leave time for them to continue schooling.

The onset of pregnancy relieved the young women from social and psychological burdens and enhanced their value in the family. However, these women were unaware of the future of the pregnancy, how pregnancy would change their bodies, what socioeconomic and physical changes they would experience and how they would look after delivering the baby when they were too young. They did not have adequate sources or well-trained people to guide them about maternal and child health. Only the older women in the family guided them through their experiences of pregnancy and childbirth. The excitement of pregnancy and satisfaction of meeting the expectations of others surpassed all concerns.

## 4. Discussion

This study aimed to find the sociocultural factors influencing adolescent motherhood among young girls in rural and less privileged areas of northern Sindh, Pakistan. The findings of this study demonstrated how poverty, marriage arrangements, the extended family system and sociocultural practices induced adolescent motherhood in the areas where social indictors of women’s empowerment were weak. Child marriage played a central role in accelerating the incidence of adolescent motherhood because parents either secured their kin group’s support or settled a dispute by marrying their young daughters. The participants’ weak position within the family made them vulnerable to these practices where they accepted the patriarchal power without questioning it. Older women living in the extended family encouraged the young married girls to conform to their reproductive roles and strengthen their family position by producing heirs. Thus, the participants aspired to be mothers since the beginning of their marriage; such preference for women’s familial roles made girls’ education less important in the villages.

South Asia has one of the highest rates of adolescent pregnancies in the world. There are various factors inducing adolescent motherhood, e.g., poverty, male dominance, child marriage, prevalent culture and limited awareness about family planning methods [39]. Adolescent pregnancy has a strong association with girls’ economic status: girls belonging to marginalized communities have higher chances of getting pregnant earlier than their counterparts belonging to economically stable households. In low-income countries, poverty plays a dual role because it influences adolescent pregnancy and worsens economic circumstances [40]. The findings of this study also highlight that teenage pregnancy and adolescent motherhood are common among underprivileged girls living in the villages. Most of the families in these villages were underprivileged, lacking economic resources. These families lived without any social support which could help them deal with health issues or pay off their loans. Therefore, a girl’s marriage was seen as a way of strengthening the kin group or settling a dispute, as happened in the case of Seema.

According to the Ghana Maternal Health Survey 2017, there are lower risks of pregnancy among women belonging to the households with the highest wealth index and among adolescents having secondary or higher education [41]. The participants in this study not only had limited education but also restricted economic opportunities. Poverty had curtailed such opportunities for them. When poverty interacted with other factors, e.g., gender roles and patriarchy, it created an adverse social environment for the young women. Moreover, the participants were socialized with rigid gender roles and were trained to fulfil their roles as service providers to keep their husbands happy and strengthen their position. Therefore, these young women considered motherhood a supreme status and struggled to achieve it soon after the marriage.

In the Pakistani context, there are various factors influencing adolescent motherhood, such as cultural practices, extended family and women’s marginalized status; child marriage is one of them [3]. Child marriage is common among rural and uneducated people and affects socially weak, poor and isolated women greatly. [42,43]. The detrimental effects of child marriage are likely to impact women’s entire lifespan, particularly girls, who are more exposed to the devastating consequences of child marriage [44].

Another factor, i.e., Child Consanguineous Marriage (CCM), leaves a devastating impact on mother and child’s health. A study investigated the combined effects of CCM in Pakistan and found a significant association between CCM and under 5 mortality [45]. The study concludes that CCM also increases the chances of infant mortality and small-sized births. The impact of consanguineous and child marriage has been investigated separately in various studies. However, the effects of consanguineous as well as child marriage, when both are combined, are not investigated yet. In our sample, 32 out of 37 adolescent girls married to their close relatives. Only 5 women had out-group marriage. Close kin group marriage was the norm in the villages. In the agrarian system of Pakistan, the family structure and kin group are strong cultural forces that control marriage decision-making. [46,47]. The patriarchal structure plays a significant role in which men decide who marries whom and when [48]. The young girls in our sample were married to their cousins, and the elders of the family arranged the marriage.

South Asian women are also known to begin maternity within 1-2 years of marriage, which is the shortest interval [49]. The findings of this study help explain the various factors and social pressures for this short interval between marriage and first pregnancy. The availability of a groom within the kin group enabled the parents to contract the marriage as soon as a girl reached puberty. These girls were unaware of the devastating consequences of CCM and happily embraced the new married life for which they were trained. The family structure played a role in influencing the participants to begin childbearing soon after the marriage.

One’s association with extended family is also associated with adolescent pregnancy and motherhood [3]. Our study highlights how the extended family members pressurized the young girls in different ways. In our sample, 33 out of 37 women lived in extended families, and 20 got pregnant between 15 and16 years of age. The mother-in-law and other older women in the family repeatedly asked the girls whether or not they had conceived. These older women would also explain to the girls the importance of motherhood and how it would strengthen their place in the family. This increased fear among the girls that if they did not conceive, their position in the family would be vulnerable. Thus, many of them were desperate to be mothers and felt safe after they succeeded in conceiving.

Modernization and globalization have brought some transition to South Asian communities, yet women’s roles as homemakers and service providers have seen little change [50,51]. The prevalent social structure in the villages of northern Sindh encouraged women to adapt to gender roles and be subordinate to men. Therefore, women internalized the system in which early marriage and maternity are considered normal. The results of this study indicate that there is a strong need to intensify education and awareness about adolescent motherhood in rural and remote areas of Sindh province. Community heads and religious leaders can play a positive role in educating people about the social, physical and health consequences of child marriage and adolescent motherhood. Findings suggest that addressing poverty and girls dropping out of school can help to increase the age at marriage and resist child marriage and adolescent motherhood in the areas where women have weak social status. Providing economic support to the parents and incentives to the girls enrolled in schools can help families deal with financial crises. Furthermore, specific and robust interventions must be explicitly introduced for rural and remote areas where young girls are more vulnerable to child marriage and adolescent pregnancy. Health providers’ role is significant in providing awareness and psychological support to young girls experiencing adolescent pregnancy and motherhood. Their counselling to the family can also strengthen parents’ role in supporting girls’ education.

### 4.1. Limitations of the study

This qualitative study presents the situation of 37 young women who belonged to a homogenous group in northern Sindh, Pakistan. Therefore, the findings of this study must be used with caution. This study documented interviews with young women only, qualitative interviews with other stakeholders, and focused group discussions with community members could have provided richer data. Nevertheless, the clearly defined qualitative methodology in this study shows that the study design can be replicated using similar methods to achieve consistent results and conclusions. Moreover, we were interested in exploring region specific factors of adolescent motherhood. A closer analysis of sociocultural factors of adolescent motherhood in various communities of Pakistan is necessary to compare and contrast similarities and differences.

## 5. Conclusion

This qualitative study analyzes the sociocultural factors inducing adolescent pregnancy and motherhood in rural and remote areas in northern Sindh, Pakistan. The study contributes to the existing knowledge about the subject by highlighting additional factors influencing adolescent motherhood in less privileged areas. Adolescent motherhood in the villages under study is a result of several underlying diverse forces, with child marriage, educational attainment and poverty being the highly associated factors. Several other social factors, e.g., extended family, limited autonomy, and little knowledge about reproductive health, accelerate the incidence of adolescent motherhood. Adolescent girls who become pregnant require more reproductive health awareness and facilities as well as attention from policymakers. Those who have weak social status and live in remote areas are more vulnerable to poor maternal health outcomes. Due to their locality, these girls were under immense pressure from social and cultural practices that further limited educational and economic opportunities. The findings of this study are important implications for understanding how to support and mentor young women who experience child marriage and become adolescent mothers. Policies and interventions must address poverty, female educational attainment and child marriage to counter adolescent motherhood. The government must initiate sex and reproductive health education to prevent child marriage and early childbearing, particularly in rural and remote communities.

## Supporting information

S1 File Coding. (DOCX)

S2 File Frequency table. (DOCX)
