# Supplementary material for: How adolescent motherhood is perceived and influenced by sociocultural factors: A sociological qualitative study of Sindh province, Pakistan
Source: PLoS One. 2025 Mar 31;20(3):e0319064. doi: 10.1371/journal.pone.0319064 (PMC11957258; doi:10.1371/journal.pone.0319064)
Supplement: S1 File — (DOCX) [file pone.0319064.s001.docx]

**Adolescent pregnancy and motherhood**

| Theme | Code | extract | Source | comments |
| --- | --- | --- | --- | --- |
| Social Pressure | Marriage arrangements/early marriage and early maternity | Cousin marriage, No pressure, early marriage is common here. | Rabia 18 (15) CM/EF | Means women have internalized the system. Early marriage and maternity is normal to them. |
|  |  | Arrange marriage, my parents selected my husband, and I agreed | Sofia 16 (16)  Out group marriage |  |
|  |  | I got married at very young age I did not know anything about being married or making my home alone at husband’s house. Too much pressure on young girls by in laws and other elderly women of society like relatives from both side. I was under pressure in first two months of my marriage as I did not seen any sign of getting pregnant but thanks to God I was relieved when my test came positive. | Nazia 20 (15) CM/NF | Young girls face pressure to get pregnancy from various side i.e. parents, in-laws, relatives, elderly women. |
|  |  | I got married when even my body was not shaped like a woman. So when I got pregnant, it was my belly only which was big, rest of my body remained same. | Seema 19 (13) FEM |  |
|  |  | In our community, a young age girl easy to gets pregnant. She gives birth to more sons and could be a normal delivery. Can do more housework, and they are more satisfying to men. If they did not get pregnant so their husband could be married to another girl to get children, she easily tolerates their cruelty. We are easily misled. We fear them. | Seema 19 (13) FEM |  |
|  |  | *It was an arrange marriage as soon as I entered my puberty age my parents stressed on my marriage. I am married to my cousin, he was earning so my parents immediately agreed. No, fairy tale, it is same common story*  *of every girl like me.* | Saima 19(17) CM/JF |  |
|  |  | We had four sisters and one brother. My father had a small job. He had a hard time raising four young daughters. It is difficult to bring up the daughters of a poor father, first, our education was terminated, then started looking for a boy. For a poor girl, the relationship also comes with great difficulty, just someone said that there are 2 brothers, they are laborers, but they are honest, and they want to marry your daughters. And our father agreed. But we had want to go to school and college. We think we live in the city. It won't all happen to us as it happened to my aunties or other cousins. | Nisa 19(16) |  |
|  | Social pressure/  Pressure faced | I became nervous during these 2months of not becoming pregnant and the other pressure on me was to birth a baby boy. | Nazia 20 (15) CM/NF |  |
|  |  | *It becomes concern of everyone after 2-3 months of marriage. Whenever any neighbourer lady visits home she first inquires me about pregnancy. I was being told since first day that home making is the sole purpose of a girl’s life. My mother told me by getting pregnant, I will have respectable position in my in-laws. From both fronts I was guided to get pregnant as soon as possible.* | Tahira 19(15) CM |  |
|  |  | Pressure is very high that girl should became mother soon after marriage . I had to convince my husband as he wasn’t ready for a child.  I told my husband that I want to became a mother as I was in pressure from family and society. I got pregnant after a month of my pregnancy. | Sofia 16 (16) |  |
|  |  | In the second month, I got pregnant so I faced pressure for the son because it was the second marriage with my husband for a son. I was afraid that if the son was not born. It will beat me. | Seema 19 (12.5) FEM |  |
|  |  | In our community, a young age girl easy to get pregnant they could be normal delivery and young girls easy to learn housework, they more satisfying to men. They have more chance to baby boy child birth. If they not get pregnant so their husband could be marry to another girl for get children. | Nisa 19 (16) |  |
|  |  | *Generally women, in our village inquire much about it. That’s why young married girl often gets in depression, thinking almost every moment when would I get pregnant. Will you believe! When I got spotting those days I was scared, if had periods unfortunately. In my case, I was forced and conditioned in a way that my husband has 6 sisters- he is sole brother to them. I was asked to bless them with baby boy now. Moreover, wherever I go I was asked why I was late in getting pregnant.* | Saima 19(17)CM/JF |  |
|  |  | There is high pressure for the girl to become mother and there is criticism against the girl to become as a mother. This kept me mentally disturbed until I got pregnant. | Sardaran 20(16) |  |
|  | Impact of Social Pressure | I used to get down in despair and hopelessness. That whenever I heard about any pregnancy I used to leave eating and I used to cry in some lone corners … | Saima 19(17)CM/JF |  |
|  |  | Sometimes, I feel fear that people rumors me that she could not able to give birth to a child, and most of the time, I avoided attending family gatherings. | Nisa 19 (16) |  |
|  | Feelings upon getting pregnant | thanks to God I was relieved when my test came positive. Happy but worried about futre due to lack of knowledge. | Nazia 20 (15) CM/NF |  |
|  |  | *Everything was new to me; I was unable to understand what is happening around. I had only one option to agree with them.* I was happy to see all those smiling faces welcoming this news. | Tahira 19 (15) CM |  |
|  |  | I felt secure, and proud to be pregnant, although financial problems made me mentally and did physically stressed. | Seema 19 (13) FEM |  |
|  |  | I felt secure, and proud to be pregnant. | Nisa 19(16) |  |
|  |  | *I was very happy that I will be delivering my first baby soon. I got this news after 6 months, I was happy that now I will be safe from taunts of other women.* | Saima 19(17) |  |
| Knowledge and Awareness |  | I had only 2 periods and was forced to get married. I got pregnant in the first month but delivered still baby in 8^th^ month. Got pregnant again in the second month and delivered 4 babies in 4 years. | Seema 19 (13) FEM |  |
|  |  | At teenage I do not know about pregnancy but mother and mother in law give information about antenatal care as food like desi ghee,milk,egg are best for pregnant | Salma 17 (17) |  |
|  | Use of medicines/multivitamins | No. medicine is not good for me and my baby’s health as said, as my husband. He did not give me a single penny so I started gagging. Whenever I felt sick I got used to Panadol only. | Seema 19 (13) FEM |  |
|  |  | I did not use any multivitamins during pregnancy. my mother said this medicine make you fatty and warm in body temperature | Nisa 19 (16) AM/JF |  |
|  |  | Yes it have been to doctor, she was very supportive as qualified fcps and from family members especially experienced. | Sofia 16 (16) |  |
|  | Use of contraceptives | My husband did not like family planning they believed that family planning is sinfulness, and he wants a baby boy. I know about FM medicine, and other protections. but we don’t follow it. This is sinful. My husband beat me if I asked him for this. | Seema 19 (13) FEM |  |
|  |  | our parents not allowed to get such types of information of family planning. They believed that all thing we learned gradually by own self, this is not well for unmarried girls.  Sometimes we discussed friends and cousins’ gathering or who already pregnant or newly married and we saw on face book how women get pregnant and bay grow inside the belly. Who we should care yourself during pregnancy. No, because my husband did not like family planning they believed that family planning is sinfulness, and we should avoid this.  I know about family planning, medicine, and other protections. but we don’t follow it. If we follow this Allah not forgive us. In my opinion in Islam we should be allowed to family planning. | Nisa 19 (16) AM/JF |  |
|  |  | *No, I have not consulted with doctor on this topic. But I have heard from elder ladies that after delivery for 40 days do not come in contact with your husband.* | Saima 19(17) |  |
| Challenges | Pregnancy management+ difficulties faced during prenatal care | The most difficult time was one month before my delivery, I was worried either it will be a normal or not social pressure was too much over me. I was tensed and had anger issues during pregnancy. I would have headache all the time. | Nazia 20 (15) CM/NF |  |
|  |  | Travelling for antenatal visit, Workload, Study gap, Financial Problems. | Sardaran 20(16) |  |
|  |  | I mostly remained upset and i was unable to do my study. | Sardaran 20(16) |  |
|  | Negotiations with household chores | They were behaving in good way because the baby who was in my womb was male. | Ayesha 19 (16) CM/EF | Cousin marriage and pregnancy with male child eased her life. |
|  |  | I did extra work as I had wanted normal delivery. | Nisa 19(16) |  |
|  |  | *The things never changed actually because the treatment from my in-laws was same nothing special. I was given the same workload. My daily routine was not changed. I used to do the same household chores before and after pregnancy. Early morning after praying and recitation I had to prepare breakfast for my husband and all other family members. Milk cattle, collect eggs and graze cattle. I used to have little time for rest then I had to prepare lunch and work goes on till sunset.*  *That only was decreased when I was unable to cope with household chores with such weak condition.* | Tahira 19 (15) |  |
|  |  | Being a pregnant teenager many challenges were faced by me to negotiate with household because I thought now it is my home and my survival is here. | Sardaran 20(16) |  |
|  |  | My husband is very supportive so he never let me down nor pressurized me to complete tasks. Movement is restricted, weight made my look tired most of times but supportive husband is a blessing, he even provides me water for my medication. | Sofia 16 (16) |  |
|  | Problems with other responsibilities |  |  |  |
|  | Weight body shape | *Yes, as it was my first pregnancy and pregnancy experience in context of body shape looked somehow was unusual to me. My weight abruptly increased, so I was reluctant to face people. I feel shy, so I gave up going out during this time. Also, after delivering baby I had stretch marks on my belly, those were scary.* | Saima 19(17) |  |
|  | Food and nutrition | *No, any addition was made to my diet. I used to take 3 times meal and take a glass of milk at night. My husband used to bring meat sometimes, but due to vomit feeling I was unable to eat that as well.* | Saima 19(17) |  |
|  | Pregnancy related symptoms |  |  |  |
|  | Financial challenges  Childbirth expenses | My husband and my Father-in-law supported to manage financial challenges | Ayesha 19 (16) CM/EF | Due to cousin marriage |
|  |  | All expenses bore by husband and FIL | Rabia 18 (15) CM/EF |  |
|  | Psychological | *Sincerely speaking sister, at that time I was haunted by only one single thought that was of gender of baby. As they were excited and used to say they want baby boy. I used to think then what if I deliver baby girl; then they shall not take care of me as they do now. I used to fear such thoughts.* | Saima 19(17) |  |
|  |  | *I had no time to ponder over my condition. I only used to work and work the whole day as I do now. Who cares to head ear to my problems? Sometimes I was unable to sleep at night thinking what would happen next. As I have heard about labour pain, I used to scare from it.* | Tahira 19(15) |  |
|  | Social challenges |  |  |  |
|  |  |  |  |  |
| Impact of teenage pregnancy | Lifestyle changes | I felt myself a mature woman,now a responsible at the age of 15. | Nazia 20 (15) CM/NF |  |
|  |  | *The changes were gradual yet drastic as I was transforming from a girl to a women now. My life changed dramatically quickly (mild laughter). When I used to look at mirror I see myself as older women now.* | Saima 19(17) |  |
|  | Education | House chores is main hurdle of education, there is no matter you are pregnant. our senior female always notified us that child care is a great responsibility, so you cannot continue your studies. | Seema 19 (13) FEM |  |
|  |  | House chores is main hurdle of education, there is no matter you are pregnant. Our senior female always notified us that child care is a great responsibility, so you cannot continue your studies. | Nisa 19 (16) |  |
|  |  | *My cousin did not want me to go outside home for school, he insisted his parents to convince my parents for our marriage. My parents could not resist longer. After I finished primary education, I was married to him. My friends are still studying, I could have studied further.* | Tahira 19(15)CM |  |
|  |  | *It did not affect my education, as I had left school earlier after primary education.* | Saima 19(17) |  |
|  |  | Yes, it affected my life totally and my inclination was to my baby. | Sardaran 20(16) |  |
|  |  | I left my education after matriculation cause can't attempt paper while pregnant and preparation is impossible. | Sofia 16 (16) |  |
|  | Health | Gained lot of weight | Nazia 20 (15) CM/NF |  |
|  |  | Sleep cycle changed fatigue, less work.  Weight increased drastically, allergies have grown on me, and vomiting, disliking favorite food. | Sofia 16 (16) |  |
|  |  | weakness, anxiety, fever in bones, headache, Dizziness, vomiting, and Not feeling hungry for whole day | Seema 19 (13) FEM |  |
|  |  | *Yes my health was largely affected as i used to remain very weak at that time-body ache, fever were common in those days.* | Saima 19(17) |  |
|  |  | Yes, i was feeling weakness, vomiting and suffering from illness. | Sardaran 20(16) |  |
|  | Food | My diet remained same as I could not afford like dry fruit and such other things, seasonal fruits added to my diet. | Nazia 20 (15) CM/NF |  |
|  |  | *No, no any changes were made in my diet. I used to eat the same as earlier* | Saima 19(17) |  |
|  | Daily routine | *My routine changed as majorly I used to make meal for the entire members of family, two times a day daily. But then I was asked to cook for single time.*  *Once, my husband had a violent behavior with me because I was reluctant to wake-up early. I told him I was feeling lethargic but he was not listening. I had to wake-up early thereafter, despite of my weak health.* | Saima 19(17) |  |
|  | Psychological issues | The psychological issues which were faced by me it was the criticism from my parents-in-laws which caused to be weight gaining and mood swing. | Sardaran 20(16) |  |
|  | Relationship with husband |  |  |  |
|  | Experience of delivering the baby | It was very good experience because it wanted to see my baby who was In my womb | Ayesha 19 (16) CM/EF |  |
|  |  | I was happy that I will be a mother and worried in a sense that I should not birth a child in this young age. The experience was horrible and wasn’t even thinking to birth another soon. | Nazia 20 (15) CM/NF |  |
|  |  | *As a woman, whatever difficulties I faced (the pain and all that) I was told that you are the only woman who is bearing such pain, health related difficulties or delivering baby. You have to learn this and have faith on All-Mighty. Despite of ill-health, I had to do all the household work. Also, due to minimal literacy I was unable to understand and solve my problems.* | Saima 19(17) |  |
|  |  | *It was a wave of fear, overwhelming my mind. Doctor told I was very weak. My mother was continuously praying for me and told me to have faith in Allah, nothing will happen. The only relief I got after having baby is also because of the existence of my son. It relieved me from stress which I never noted I had. Now at least I had someone most dear to me, near to me.* | Tahira 19(15) |  |
|  | Outcome of the pregnancy | First still birth in 8^th^ month. Childbearing at a young age is very dangerous and many girls have died. My bleeding had not stopped after delivery. The first child died in the womb. The husband had intercourse in the eighth month due to which the pains started. | Seema 19 (13) EM | They only know through their experiences. |
|  |  | My cousin was also married at the age of 13, her child was born prematurely, the child survived but the mother died on the way. And after 20 days the child also died because it is said that she had a demon. | Seema 19 (13) EM |  |
|  |  | Their mother-in-law believed that house chores may provide support for normal deliveries, therefore a load of house chores grew heavier every day. Due to their precarious health circumstances, they had unable to handle the delivery, and her sister's baby died while being delivered. and during the second baby delivery, she along her baby died. | Nisa 19 (16) |  |
|  |  | *My doctor also told me that my baby is weak due to mental stress and inssufficient diet.* | Saima 19(17) |  |
|  | Life changes after the delivery |  |  |  |
|  | Violence |  |  |  |
| Social support | Family support | It was very positive role from family side to new change in my life they never felt to me that i’m alone who is fighting the battle of survival. | Ayesha 19 (16) CM/EF |  |
|  |  | *It was hard to do house chores with heavy belly weight. I used to sit down and start doing work again. Moreover, wife of my brother-in-law helped me, whenever I felt down. Their behavior was neutral. There are kids of my brother-in-law. Thus, it was not the first happy news there. It was considered normal. It was only in last month my mother-in-law paid some attention to me. Moreover, it was wife of my brother in law who was somehow supportive.* | Tahira 19(15)  See her interview for more |  |
|  |  | During first 7 months everything seemed normal to me and last two months specially the last month was difficult for me. My sisters, mother and dai supported me. | Nazia 20 (15) CM/NF |  |
|  |  | Did extra work, did not receive anchoresy support due to the family feud of brother. My in-law abused, taunted abd beat me. They didn't even let me go home. My parents did not try to meet they did not want to listen to me. | Seema 19 (13) EM | So the basis of marriage and feuds decide the extent of care received during the pregnancy. |
|  |  | My mother-in-law said, you don't behave like a child now you've grown up. This is a part of women's lives and made for it. | Nisa 19(16) |  |
|  |  | There was no moral support during my pregnancy because whenever i was looking to anyone for moral support i was denied. | Sardaran 20(16) |  |
|  |  | First there was no support to me from my husband but when he heard the baby is male whom i’m going to give birth so he supported me alot because he needed male heir. | Sardaran 20(16) |  |
|  |  | *My husband and mother-in-law often used to console me and they usually advised me to take care of myself these days – take meal on time, do not carry any huge weight etc. It was hard as I feel very shy to see myself in such condition. Moreover, it was my weak health condition that was painful and often unbearable.*  *They just used to help and assist me in my daily work/chores that the burden of work could deteriorate my health and consequently their son/grandson health* | Saima 19(17) |  |
|  |  | First there was no support to me from my husband but when he heard the baby is male whom i’m going to give birth so he supported me a lot because he needed male heir. | Sardaran (20)16 |  |
|  | Husband’s support to face the challenges |  | Rabia 18 (15) CM/EF | Supported by husband through all stages of pregnancy. |
|  | Husband’s support for visiting doctor |  | See sofia |  |
|  | Role of healthcare provider |  |  |  |
|  | Support from others |  |  |  |
|  | Family’s support after child birth | I had to be responsible after baby I could not attend events in my neighborhood until my mother in law take care of my baby, I was too much bounded around my daughter. My mother often used to live at my house to take care of me and my baby she supported me in my all pregnancies. | Nazia 20 (15) CM/NF |  |
|  |  | *The only support I had from my mother in law was he used to take care of my new born when I was busy doing work.* | Tahira 19(15) |  |
|  |  |  |  |  |
|  |  |  |  |  |
