# Supplementary material for: How adolescent motherhood is perceived and influenced by sociocultural factors: A sociological qualitative study of Sindh province, Pakistan
Source: PLoS One. 2025 Mar 31;20(3):e0319064. doi: 10.1371/journal.pone.0319064 (PMC11957258; doi:10.1371/journal.pone.0319064)
Supplement: S2 File — (DOCX) [file pone.0319064.s002.docx]

**Demographic data**

| **Variables Frequency** |
| --- |
| **Age of the respondents** |
| 15-16 09 |
| 17-18 08 |
| 19-20 20 |
| **Age at marriage** |
| 13-14 07 |
| 15-16 22 |
| 17-18 08 |
| **Husband’s age at marriage** |
| 15-18 04 |
| 19-22 11 |
| 23-26 09 |
| 27-30 08 |
| 31 & above 05 |
| **Age at the first pregnancy** |
| 15-16 20 |
| 17-18 17 |
| **No of children** |
| 00 01 |
| 01 08 |
| 02 22 |
| 03 06 |
| **Respondents’ education level** |
| None 16 |
| Primary 08 |
| Secondary 03 |
| Higher Secondary 02 |
| Intermediate 06 |
| Graduate 02 |
|  |
| **Husbands’ education level** |
| None 13 |
| Primary 05 |
| Secondary 01 |
| Higher secondary 03 |
| Intermediate 02 |
| Graduate 05 |
| Master 08 |
| **Fathers’ education level** |
| None 21 |
| Primary 04 |
| Secondary 03 |
| Higher secondary 05 |
| Intermediate 04 |
| Graduate 00 |
| Master 00 |
| **Mothers’ education level** |
| None 32 |
| Primary 02 |
| Secondary 01 |
| Higher secondary 00 |
| Intermediate 02 |
| Graduate 00 |
| Master 00 |
| **Monthly income of Husband** |
| 5k-10k 09 |
| 11k-15k 12 |
| 16k-20k 07 |
| 20k-25k 03 |
| 26k-30k 04 |
| 31k & above 02 |
| **Family Type** |
| Extended 33 |
| Nuclear 04 |
| **Marriage type** |
| Cousin marriage 16 |
| Exchange marriage 12 |
| Out of group marriage 05 |
| Marriage for bride price 04 |
| **Number of family members** |
| 02-04 03 |
| 05-07 11 |
| 08-10 13 |
| 11 & above 10 |
| **Knowledge about legal age of marriage** |
| Yes 09 |
| No 23 |
| To some extent 05 |
| **Whether it was your planned pregnancy** |
| Yes 10 |
| No 27 |
| **Health information received during first pregnancy** |
| Yes 15 |
| No 18 |
| To some extent 04 |
| **Information about pregnancy care given by family members** |
| Yes 14 |
| No 22 |
| To some extent 01 |
| **Feelings during first pregnancy** |
| Disturbed 15 |
| Distressed 11 |
| Happy 11 |
| **Number of time scanning during pregnancy** |
| None 18 |
| 1-2 14 |
| 3-4 03 |
| 5 & above 02 |
| **Blood test during pregnancy** |
| Yes 22 |
| No 15 |
| **Knowledge about different method of family planning** |
| Yes 10 |
| No 20 |
| To some extent 07 |
| **Knowledge about harmful impacts of teenage pregnancy** |
| Yes 11 |
| No 21 |
| To some extent 05 |
| **Knowledge about family planning upon marriage** |
| Yes 07 |
| No 27 |
| To some extent 03 |
| **Do you know average age of girls marry in your area?** |
| Yes 26 |
| No 11 |
| **How often did you visit health expert during your pregnancy?** |
| Usually 10 |
| Occasionally 19 |
| Never visited 08 |
| **Use of multivitamin during pregnancy** |
| Yes 17 |
| No 20 |
| **Where did you deliver your first baby?** |
| Home 18 |
| Private clinic 08 |
| Public clinic 11 |
| **Who delivered your first baby?** |
| Dai 10 |
| Skilled birth attendant 18 |
| Doctor 09 |
| **Did you ever discuss about pregnancy prevention with family or friends?** |
| Yes 12 |
| No 25 |
